# Prevention and Control of Seasonal Influenza with Vaccines: Recommendations of the Advisory Committee on Immunization Practices (ACIP) — United States, 2014–15 Influenza Season

**Published:** 2014-08-15

**Authors:** Lisa A. Grohskopf, Sonja J. Olsen, Leslie Z. Sokolow, Joseph S. Bresee, Nancy J. Cox, Karen R. Broder, Ruth A. Karron, Emmanuel B. Walter

**Affiliations:** 1 Influenza Division, National Center for Immunization and Respiratory Diseases, CDC; 2 Immunization Safety Office, National Center for Emerging and Zoonotic Infectious Diseases, CDC; 3 Johns Hopkins University; 4 Duke University School of Medicine

This report updates the 2013 recommendations by the Advisory Committee on Immunization Practices (ACIP) regarding use of seasonal influenza vaccines (1). Updated information for the 2014–15 influenza season includes 1) antigenic composition of U.S. seasonal influenza vaccines; 2) vaccine dose considerations for children aged 6 months through 8 years; and 3) a preference for the use, when immediately available, of live attenuated influenza vaccine (LAIV) for healthy children aged 2 through 8 years, to be implemented as feasible for the 2014–15 season but not later than the 2015–16 season. Information regarding issues related to influenza vaccination not addressed in this report is available in the 2013 ACIP seasonal influenza recommendations (1).

*Recommendations for routine use of vaccines in children, adolescents, and adults are developed by the Advisory Committee on Immunization Practices (ACIP). ACIP is chartered as a federal advisory committee to provide expert external advice and guidance to the Director of the Centers for Disease Control and Prevention (CDC) on use of vaccines and related agents for the control of vaccine-preventable diseases in the civilian population of the United States. Recommendations for routine use of vaccines in children and adolescents are harmonized to the greatest extent possible with recommendations made by the American Academy of Pediatrics (AAP), the American Academy of Family Physicians (AAFP), and the American College of Obstetrics and Gynecology (ACOG). Recommendations for routine use of vaccines in adults are harmonized with recommendations of AAFP, ACOG, and the American College of Physicians (ACP). ACIP recommendations adopted by the CDC Director become agency guidelines on the date published in the* Morbidity and Mortality Weekly Report (MMWR)*. Additional information regarding ACIP is available at http://www.cdc.gov/vaccines/acip.*

For recommendations pertaining to use of influenza vaccines in children, ACIP reviewed data on the relative efficacy and safety of LAIV and inactivated influenza vaccines (IIVs). An adapted version of the Grading of Recommendations Assessment, Development and Evaluation (GRADE) approach was used to rate the quality of the evidence (2). Evidence summary tables and assessment of risk and benefits are available at http://www.cdc.gov/vaccines/acip/recs/grade/table-refs.html. Information in this report reflects discussion during public meetings of ACIP on February 26, 2014, and June 25, 2014. Meeting minutes, information on ACIP membership, and information on conflicts of interest are available at http://www.cdc.gov/vaccines/acip/meetings/meetings-info.html. Modifications were made during review at CDC to update and clarify wording. Any updates will be posted at http://www.cdc.gov/flu.

## Groups Recommended for Vaccination and Timing of Vaccination

Routine annual influenza vaccination is recommended for all persons aged ≥6 months who do not have contraindications. Vaccination optimally should occur before onset of influenza activity in the community. Health care providers should offer vaccination soon after vaccine becomes available (by October, if possible). Vaccination should be offered as long as influenza viruses are circulating. Children aged 6 months through 8 years who require 2 doses (see “Vaccine Dose Considerations for Children Aged 6 Months through 8 Years”) should receive their first dose as soon as possible after vaccine becomes available, and the second dose ≥4 weeks later. To avoid missed opportunities for vaccination, providers should offer vaccination during routine health care visits and hospitalizations when vaccine is available.

Antibody levels induced by vaccine decline postvaccination (3–6). Although a 2008 literature review found no clear evidence of more rapid decline among the elderly (7), a 2010 study noted a statistically significant decline in titers 6 months postvaccination among persons aged ≥65 years (although titers still met European Medicines Agency levels considered adequate for protection) (6). A case-control study conducted in Navarre, Spain, during the 2011–12 season revealed a decline in vaccine effectiveness primarily affecting persons aged ≥65 years (8). Although delaying vaccination might permit greater immunity later in the season, deferral might result in missed opportunities to vaccinate and difficulties in vaccinating a population within a limited time. Vaccination programs should balance maximizing likelihood of persistence of vaccine-induced protection through the season with avoiding missed opportunities to vaccinate or vaccinating after influenza virus circulation begins.

## Influenza Vaccine Composition for the 2014–15 Season

For 2014–15, U.S.-licensed influenza vaccines will contain the same vaccine virus strains as those in the 2013–14 vaccine. Trivalent influenza vaccines will contain hemagglutinin (HA) derived from an A/California/7/2009 (H1N1)-like virus, an A/Texas/50/2012 (H3N2)-like virus, and a B/Massachusetts/2/2012-like (Yamagata lineage) virus. Quadrivalent influenza vaccines will contain these antigens, and also a B/Brisbane/60/2008-like (Victoria lineage) virus (9).

## Available Vaccine Products and Indications

Various influenza vaccine products are anticipated to be available during the 2014–15 season (Table). These recommendations apply to all licensed influenza vaccines used within Food and Drug Administration–licensed indications. Differences between ACIP recommendations and labeled indications have been noted (Table).

## Vaccine Dose Considerations for Children Aged 6 Months through 8 Years

Children aged 6 months through 8 years require 2 doses of influenza vaccine (administered ≥4 weeks apart) during their first season of vaccination to optimize immune response (10,11). In one study conducted over two seasons during which the influenza A(H1N1) vaccine virus strain did not change but the B antigen did change, unprimed children aged 10 through 24 months who received 1 dose of IIV during the fall of each season had similar responses to the unchanged A(H1N1) virus antigen and to the drifted A(H3N2) virus antigen, compared with children aged 6 through 24 months who received 2 doses of the same IIV during the latter season; however, the first group had significantly lower responses to the B antigen (12). In determining the appropriate number of doses, previous receipt of vaccine containing 2009 influenza A(H1N1) pandemic antigen (included in monovalent pandemic vaccine during 2009–10 and in seasonal influenza vaccines since the 2010–11 season) also should be considered. In addition, because the strains contained in the 2014–15 seasonal influenza vaccines are identical to those contained in the 2013–14 vaccines, only 1 dose is required for any child aged 6 months through 8 years who previously received ≥1 dose of 2013–14 seasonal influenza vaccine.

**FIGURE 1:**
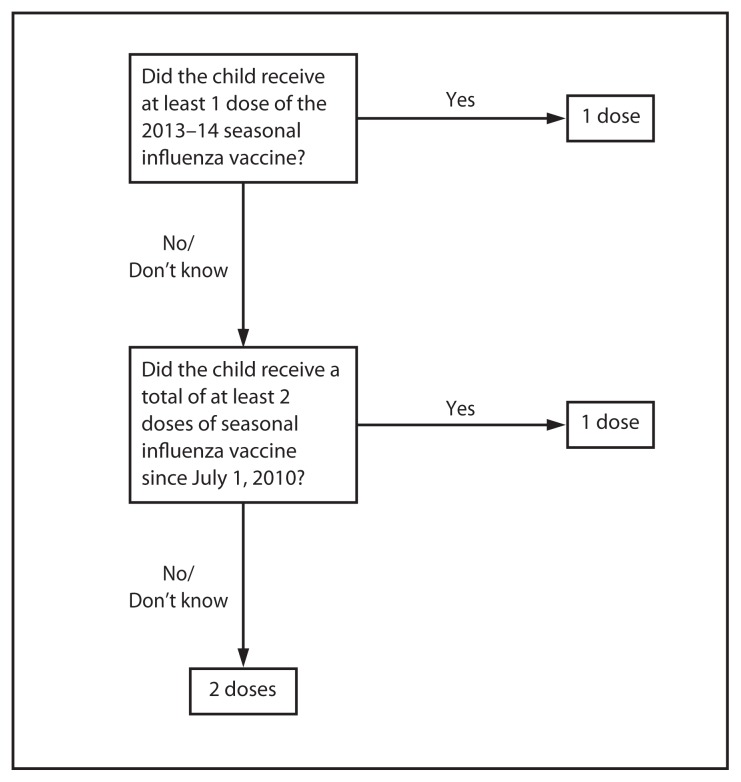
Influenza vaccine dosing algorithm for children aged 6 months through 8 years — Advisory Committee on Immunization Practices, United States, 2014–15 influenza season* * For simplicity, this algorithm takes into consideration only doses of seasonal influenza vaccine received since July 1, 2010, to determine the number of doses needed for the 2014–15 season. As an alternative approach in settings where vaccination history from before July 1, 2010, is available, if a child aged 6 months through 8 years is known to have received either 1) at least 1 dose of 2013–14 seasonal influenza vaccine, or 2) at least two seasonal influenza vaccines during any previous season, and at least 1 dose of a 2009(H1N1)–containing vaccine (i.e., seasonal vaccine since 2010–11 or the monovalent 2009[H1N1] vaccine), then the child needs only 1 dose for 2014–15. Using this approach, children aged 6 months through 8 years need only 1 dose of vaccine for 2014–15 if they have received any of the following: 1) at least 1 dose of 2013–14 seasonal influenza vaccine; or 2) 2 or more doses of seasonal influenza vaccine since July 1, 2010; or 3) 2 or more doses of seasonal influenza vaccine before July 1, 2010, and 1 or more doses of monovalent 2009(H1N1) vaccine; or 4) 1 or more doses of seasonal influenza vaccine before July 1, 2010, and 1 or more doses of seasonal influenza vaccine since July 1, 2010. Children in this age group for whom one of these conditions is not met require 2 doses for 2014–15. ^†^ Doses should be administered at least 4 weeks apart.

Two approaches are recommended for determination of the necessary doses for the 2014–15 season; both are acceptable. The first approach (Figure 1) considers only doses of seasonal influenza vaccine received since July 1, 2010. Where adequate vaccination history from before the 2010–11 season is available, the second approach (Figure 1 [footnote]) may be used.

## Considerations for the Use of Live Attenuated Influenza Vaccine and Inactivated Influenza Vaccine when Either is Available and Appropriate

Both LAIV and IIV have been demonstrated to be effective in children and adults. In adults, most comparative studies have demonstrated either that LAIV and IIV were of similar efficacy or that IIV was more efficacious (13–18). However, several studies have demonstrated superior efficacy of LAIV in children. A randomized controlled trial conducted among 7,852 children aged 6–59 months demonstrated a 55% reduction in culture-confirmed influenza among children who received LAIV compared with those who received IIV. LAIV efficacy was higher than that of IIV against both antigenically drifted and well-matched influenza viruses (19). Compared with IIV, LAIV provided 32% increased protection in preventing culture-confirmed influenza in children and adolescents aged 6–17 years with asthma (20) and 52% increased protection in children aged 6–71 months who had previously experienced recurrent respiratory tract infections (21).

ACIP reviewed the evidence pertaining to the relative efficacy of LAIV and IIV for healthy children, and concluded that LAIV is more efficacious than IIV against laboratory-confirmed influenza among younger children (based on studies including children aged 6 through 71 months), with overall moderate quality of evidence. Risks for harms assessed (including fever, wheezing, and serious adverse events) appear to be similar for LAIV and IIV. Data pertaining to relative efficacy are more limited in older children and teens. There are insufficient data to determine at what age or with how many successive seasons of vaccination the relatively greater efficacy of LAIV diminishes in children aged 6 through 18 years.

For children and adults with chronic medical conditions conferring a higher risk for influenza complications, data on the relative safety and efficacy of LAIV and IIV are limited. A study of LAIV and IIV among children aged 6 through 17 years with asthma noted no significant difference in wheezing events after LAIV (20). Available data are insufficient to determine the level of severity of asthma for which administration of LAIV would be inadvisable.

For 2014–15, ACIP recommends the following:

1. All persons aged ≥6 months should receive influenza vaccine annually. Influenza vaccination should not be delayed to procure a specific vaccine preparation if an appropriate one is already available.
2. When immediately available, LAIV should be used for healthy children aged 2 through 8 years who have no contraindications or precautions (Category A). If LAIV is not immediately available, IIV should be used. Vaccination should not be delayed to procure LAIV. The age of 8 years is selected as the upper age limit for this recommendation based on demonstration of superior efficacy of LAIV (ages 2 to 6 years), and for programmatic consistency (8 years is the upper age limit for receipt of 2 doses of influenza vaccine in a previously unvaccinated child). This recommendation should be implemented for the 2014–15 season as feasible, but not later than the 2015–16 season.
3. LAIV should not be used in the following populations:
  — Persons aged <2 years or >49 years;
  — Those with contraindications listed in the package insert:
    ○ Children aged 2 through 17 years who are receiving aspirin or aspirin-containing products;
    ○ Persons who have experienced severe allergic reactions to the vaccine or any of its components, or to a previous dose of any influenza vaccine;
  — Pregnant women;
  — Immunosuppressed persons;
  — Persons with a history of egg allergy;
  — Children aged 2 through 4 years who have asthma or who have had a wheezing episode noted in the medical record within the past 12 months, or for whom parents report that a health care provider stated that they had wheezing or asthma within the last 12 months (Table [footnote]). [For those aged ≥5 years with asthma, recommendations are described in item 4 of this list];
  — Persons who have taken influenza antiviral medications within the previous 48 hours.
4. In addition to the groups for whom LAIV is not recommended above, the “Warnings and Precautions” section of the LAIV package insert indicates that persons of any age with asthma might be at increased risk for wheezing after administration of LAIV (22), and notes that the safety of LAIV in persons with other underlying medical conditions that might predispose them to complications after wild-type influenza infection (e.g., chronic pulmonary, cardiovascular [except isolated hypertension], renal, hepatic, neurologic, hematologic, or metabolic disorders [including diabetes mellitus] ( 1 )) has not been established. These conditions, in addition to asthma in persons aged ≥5 years, should be considered precautions for the use of LAIV.
5. Persons who care for severely immunosuppressed persons who require a protective environment should not receive LAIV, or should avoid contact with such persons for 7 days after receipt, given the theoretical risk for transmission of the live attenuated vaccine virus.

## Influenza Vaccination of Persons with a History of Egg Allergy

With the exceptions of trivalent recombinant influenza vaccine (RIV3 [FluBlok], Protein Sciences) and cell culture-based inactivated influenza vaccine (ccIIV3 [Flucelvax], Novartis), currently available influenza vaccines are prepared by propagation of virus in embryonated chicken eggs. A review of published data (including data on 4,172 patients, 513 of whom were reported to have a history of severe allergic reaction to egg) noted that no occurrences of anaphylaxis were reported, although some milder reactions did occur (23), suggesting that severe allergic reactions to egg-based influenza vaccines are unlikely. On this basis, some guidance recommends that no additional measures are needed when administering influenza vaccine to egg-allergic persons (24). However, occasional cases of anaphylaxis in egg-allergic persons have been reported to the Vaccine Adverse Event Reporting System (VAERS) after administration of influenza vaccine (25,26). In published studies, vaccines containing as much as 0.7 *μ*g/0.5 mL of ovalbumin have been tolerated (27,28); however, a threshold below which no reactions would be expected is not known (27). Among IIVs for which ovalbumin content was disclosed during the 2011–12 through 2013–14 seasons, the reported maximum amounts were ≤1 *μ*g/0.5 mL dose. Ovalbumin is not directly measured for Flucelvax; it is estimated by calculation from the initial content in the reference virus strains to contain less than 5×10-8 *μ*g of total egg protein per 0.5mL dose, of which ovalbumin is a fraction (Novartis, personal communication, 2013). FluBlok is considered egg-free. However, neither Flucelvax nor FluBlok are licensed for use in children aged <18 years.

**FIGURE 2:**
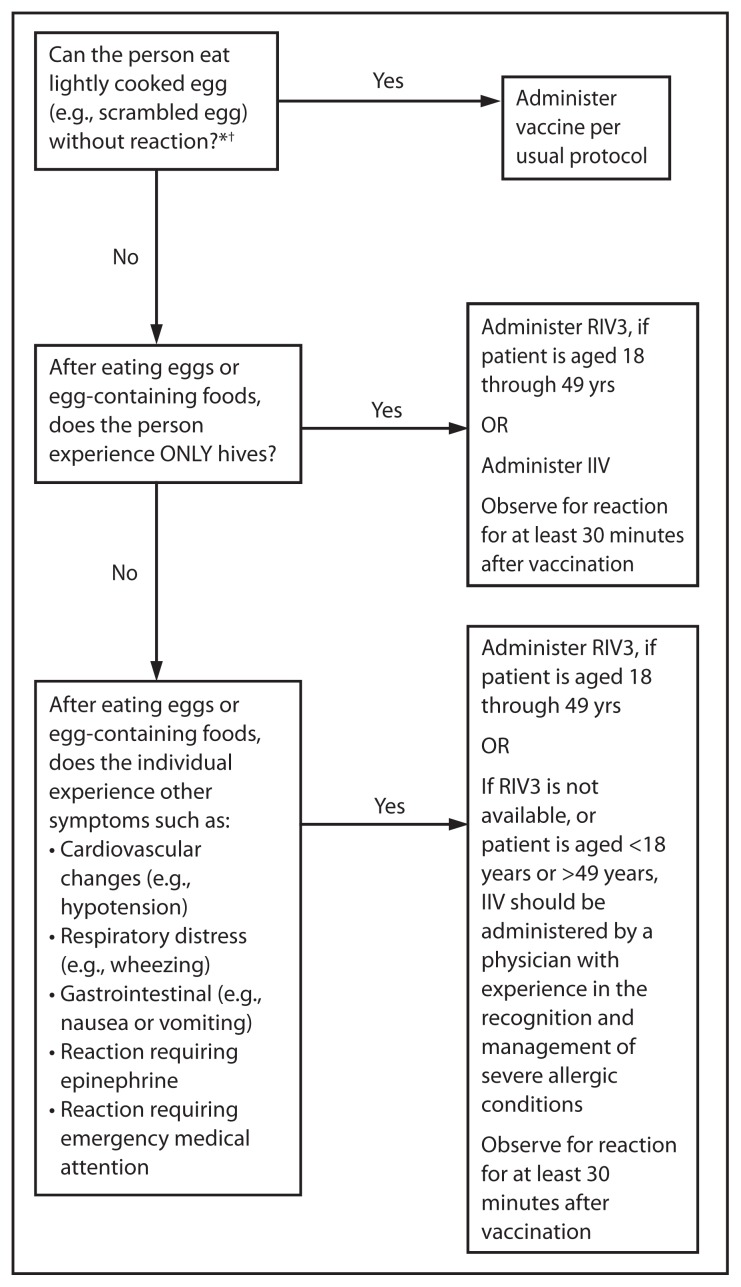
Recommendations regarding influenza vaccination of persons who report allergy to eggs — Advisory Committee on Immunization Practices, United States, 2014–15 influenza season **Abbreviations:** IIV = inactivated influenza vaccine; RIV3 = recombinant influenza vaccine, trivalent. * Persons with egg allergy might tolerate egg in baked products (e.g., bread or cake). Tolerance to egg-containing foods does not exclude the possibility of egg allergy (Erlewyn-Lajeunesse M, Brathwaite N, Lucas JS, Warner JO. Recommendations for the administration of influenza vaccine in children allergic to egg. BMJ 2009;339:b3680). ^†^ For persons who have no known history of exposure to egg, but who are suspected of being egg-allergic on the basis of previously performed allergy testing, consultation with a physician with expertise in the management of allergic conditions should be obtained before vaccination. Alternatively, RIV3 may be administered if the recipient is aged 18 through 49 years.

ACIP recommends the following:

1. Persons with a history of egg allergy who have experienced only hives after exposure to egg should receive influenza vaccine. Because relatively few data are available for use of LAIV in this setting, IIV or trivalent recombinant influenza vaccine (RIV3) should be used. RIV3 may be used for persons aged 18 through 49 years who have no other contraindications. However, IIV (egg- or cell-culture based) may also be used, with the following additional safety measures (Figure 2):
  — Vaccine should be administered by a health care provider who is familiar with the potential manifestations of egg allergy; and
  — Vaccine recipients should be observed for ≥30 minutes for signs of a reaction after administration of each vaccine dose.
2. Persons who report having had reactions to egg involving such symptoms as angioedema, respiratory distress, lightheadedness, or recurrent emesis; or who required epinephrine or another emergency medical intervention, may receive RIV3 if they are aged 18 through 49 years and there are no other contraindications. If RIV3 is not available or the recipient is not within the indicated age range, IIV should be administered by a physician with experience in the recognition and management of severe allergic conditions (Figure 2).
3. Regardless of allergy history, all vaccines should be administered in settings in which personnel and equipment for rapid recognition and treatment of anaphylaxis are available (29).
4. Persons who are able to eat lightly cooked egg (e.g., scrambled egg) without reaction are unlikely to be allergic. Egg-allergic persons might tolerate egg in baked products (e.g., bread or cake). Tolerance to egg-containing foods does not exclude the possibility of egg allergy. Egg allergy can be confirmed by a consistent medical history of adverse reactions to eggs and egg-containing foods, plus skin and/or blood testing for immunoglobulin E directed against egg proteins (30).
5. For persons with no known history of exposure to egg, but who are suspected of being egg-allergic on the basis of previously performed allergy testing, consultation with a physician with expertise in the management of allergic conditions should be obtained before vaccination (Figure 2). Alternatively, RIV3 may be administered if the recipient is aged 18 through 49 years.
6. A previous severe allergic reaction to influenza vaccine, regardless of the component suspected of being responsible for the reaction, is a contraindication to future receipt of the vaccine.

What is currently recommended?

The Advisory Committee on Immunization Practices (ACIP) recommends that all persons aged ≥6 months without contraindications receive annual vaccinations for protection against seasonal influenza. A number of different seasonal influenza vaccine formulations are available, some of which are licensed for specific age groups or are more appropriate than others for persons with certain medical conditions.

Why are the recommendations being modified now?

CDC and ACIP issue guidance on seasonal influenza vaccination annually. The current document contains updated recommendations made by ACIP in February and June 2014, to be effective for the 2014–15 season.

What are the new recommendations?

Annual influenza vaccination is recommended for all persons aged 6 months and older, as has been recommended since the 2010–11 influenza season. This guidance contains some new information. Because the virus composition of the 2014–15 seasonal influenza vaccine is the same as it was for the 2013–14 season, children aged 6 months through 8 years need only 1 dose of vaccine in 2014–15 if they received ≥1 dose of 2013–14 seasonal influenza vaccine, regardless of previous vaccination history. Other information regarding determining whether 1 or 2 doses are needed is discussed in this report. There are also new recommendations regarding the use of live attenuated influenza vaccine (LAIV) for healthy children aged 2 through 8 years. When immediately available, LAIV should be used for healthy children aged 2 years through 8 years who have no contraindications or precautions. However, inactivated influenza vaccine (IIV) should be used if LAIV is not immediately available. Vaccination should not be delayed to get LAIV.

## Figures and Tables

**TABLE:** Influenza vaccines — United States, 2014–15 influenza season*

Trade name	Manufacturer	Presentation	Mercury content from thimerosal (*μ*g Hg/0.5 mL)	Ovalbumin content (*μ*g/0.5mL)	Age indications	Route
**Inactivated influenza vaccine, quadrivalent (IIV4), standard dose**						
*Contraindications* * *: Severe allergic reaction to any component of the vaccine, including egg protein, or after previous dose of any influenza vaccine.*						
*Precautions* * *: Moderate to severe illness with or without fever; history of Guillain-Barré syndrome within 6 weeks of receipt of influenza vaccine.*						
Fluarix Quadrivalent	GlaxoSmithKline	0.5 mL single-dose prefilled syringe	—	≤0.05	≥3 yrs	IM†
FluLaval Quadrivalent	ID Biomedical Corporation of Quebec (distributed by GlaxoSmithKline)	0.5 mL single-dose prefilled syringe	—	≤0.3	≥3 yrs	IM†
		5.0 mL multidose vial	<25	≤0.3	≥3 yrs	IM†
Fluzone Quadrivalent	Sanofi Pasteur	0.25 mL single-dose prefilled syringe	—	§§§	6–35 mos	IM†
		0.5 mL single-dose prefilled syringe	—	§§§	≥36 mos	IM†
		0.5 mL single-dose vial	—	§§§	≥36 mos	IM†
		5.0 mL multidose vial	25	§§§	≥6 mos	IM†
**Inactivated influenza vaccine, trivalent (IIV3), standard dose**						
*Contraindications* * *: Severe allergic reaction to any component of the vaccine, including egg protein, or after previous dose of any influenza vaccine.*						
*Precautions* * *: Moderate to severe illness with or without fever; history of Guillain-Barré syndrome within 6 weeks of receipt of influenza vaccine.*						
Afluria	bioCSL	0.5 mL single-dose prefilled syringe	—	<1	≥9 yrs***	IM†
		5.0 mL multidose vial	24.5	<1	≥9 yrs***	IM†
Fluarix	GlaxoSmithKline	0.5 mL single-dose prefilled syringe	—	≤0.05	≥3 yrs	IM†
FluLaval	ID Biomedical Corporation of Quebec (distributed by GlaxoSmithKline)	0.5 mL single-dose prefilled syringe	—	≤0.3	≥3 yrs	IM†
		5.0 mL multidose vial	<25	≤0.3	≥3 yrs	IM†
Fluvirin	Novartis Vaccines and Diagnostics	0.5 mL single-dose prefilled syringe	≤1	≤1	≥4 yrs	IM†
		5.0 mL multidose vial	25	≤1	≥4 yrs	IM†
Fluzone	Sanofi Pasteur	0.5 mL single-dose prefilled syringe	—	§§§	≥36 mos	IM†
		5.0 mL multidose vial	25	§§§	≥6 mos	IM†
Fluzone Intradermal§	Sanofi Pasteur	0.1 mL prefilled microinjection system	—	§§§	18–64 yrs	ID**
**Inactivated influenza vaccine, trivalent, standard dose, cell culture-based (ccIIV3)**						
*Contraindications* * *: Severe allergic reaction to any component of the vaccine, including egg protein, or after previous dose of any influenza vaccine.*						
*Precautions* * *: Moderate to severe illness with or without fever; history of Guillain-Barré syndrome within 6 weeks of receipt of influenza vaccine.*						
Flucelvax	Novartis Vaccines and Diagnostics	0.5 mL single-dose prefilled syringe	—	†††	≥18 yrs	IM†
**Inactivated influenza vaccine, trivalent (IIV3), high dose**						
*Contraindications* * *: Severe allergic reaction to any component of the vaccine, including egg protein, or after previous dose of any influenza vaccine.*						
*Precautions* * *: Moderate to severe illness with or without fever; history of Guillain-Barré syndrome within 6 weeks of receipt of influenza vaccine.*						
Fluzone High-Dose††	Sanofi Pasteur	0.5 mL single-dose prefilled syringe	—	§§§	≥65 yrs	IM†
**Recombinant influenza vaccine, trivalent (RIV3)**						
*Contraindications* * *: Severe allergic reaction to any component of the vaccine.*						
*Precautions* * *: Moderate to severe illness with or without fever; history of Guillain-Barré syndrome within 6 weeks of receipt of influenza vaccine.*						
FluBlok	Protein Sciences	0.5 mL single-dose vial	—	0	18–49 yrs	IM†
**Live attenuated influenza vaccine, quadrivalent (LAIV4)**						
*Contraindications* * *: Severe allergic reaction to any component of the vaccine, including egg protein, or after previous dose of any influenza vaccine.*						
*Concomitant use of aspirin or aspirin-containing medications in children and adolescents.*						
*In addition, ACIP recommends LAIV4 not be used for pregnant women, immunosuppressed persons, persons with egg allergy, and children aged 2–4 years who have asthma or who have had a wheezing episode noted in the medical record within the past 12 months, or for whom parents report that a health care provider stated that they had wheezing or asthma within the last 12 months.*						
*LAIV should not be administered to persons who have taken influenza antiviral medications within the previous 48 hours. Persons who care for severely immunosuppressed persons who require a protective environment should not receive LAIV, or should avoid contact with such persons for 7 days after receipt.*						
*Precautions* * *: Moderate to severe illness with or without fever.*						
*History of Guillain-Barré syndrome within 6 weeks of receipt of influenza vaccine.*						
*Asthma in persons aged 5 years and older.*						
*Medical conditions which might predispose to higher risk for complications attributable to influenza.*						
FluMist Quadrivalent§§	MedImmune	0.2 mL single-dose prefilled intranasal sprayer	—	<0.24 (per 0.2mL)	2–49 yrs	IN

**Abbreviations:** IM = intramuscular; ID = intradermal; IN = intranasal; ACIP = Advisory Committee on Immunization Practices.

* Immunization providers should check Food and Drug Administration–approved prescribing information for 2014–15 influenza vaccines for the most complete and updated information, including (but not limited to) indications, contraindications, warnings, and precautions. Package inserts for U.S.-licensed vaccines are available at http://www.fda.gov/biologicsbloodvaccines/vaccines/approvedproducts/ucm093833.htm.

† For adults and older children, the recommended site of vaccination is the deltoid muscle. The preferred site for infants and young children is the anterolateral aspect of the thigh. Specific guidance regarding site and needle length for intramuscular administration can be found in ACIP’s *General Recommendations on Immunization* (available at http://www.cdc.gov/mmwr/preview/mmwrhtml/rr6002a1.htm).

§ Trivalent inactivated vaccine, intradermal: A 0.1-mL dose contains 9 *μ*g of each vaccine antigen (27 *μ*g total).

** The preferred site is over the deltoid muscle. Fluzone Intradermal is administered using the delivery system included with the vaccine.

†† Trivalent inactivated vaccine, high-dose: A 0.5-mL dose contains 60 *μ*g of each vaccine antigen (180 *μ*g total).

§§ FluMist is shipped refrigerated and stored in the refrigerator at 35°F–46°F (2°C–8°C) after arrival in the vaccination clinic. The dose is 0.2 mL divided equally between each nostril. Health care providers should consult the medical record, when available, to identify children aged 2 through 4 years with asthma or recurrent wheezing that might indicate asthma. In addition, to identify children who might be at greater risk for asthma and possibly at increased risk for wheezing after receiving LAIV, parents or caregivers of children aged 2 through 4 years should be asked, “In the past 12 months, has a health care provider ever told you that your child had wheezing or asthma?” Children whose parents or caregivers answer “yes” to this question and children who have asthma or who had a wheezing episode noted in the medical record within the past 12 months should not receive FluMist.

*** Age indication per package insert is ≥5 years; however, ACIP recommends Afluria not be used in children aged 6 months through 8 years because of increased risk for febrile reactions noted in this age group with bioCSL’s 2010 Southern Hemisphere IIV3. If no other age-appropriate, licensed inactivated seasonal influenza vaccine is available for a child aged 5 through 8 years who has a medical condition that increases the child’s risk for influenza complications, Afluria can be used; however, providers should discuss with the parents or caregivers the benefits and risks of influenza vaccination with Afluria before administering this vaccine. Afluria may be used in persons aged ≥9 years.

††† Information not included in package insert. Estimated to contain <50 femtograms (5×10-8 *μ*g) of total egg protein (of which ovalbumin is a fraction) per 0.5 mL dose of Flucelvax.

§§§ Available upon request from Sanofi Pasteur (telephone: 1-800-822-2463; e-mail: mis.emails@sanofipasteur.com).
